# Pregnancy outcomes and management in lung and heart transplant recipients: A systematic review

**DOI:** 10.1016/j.jhlto.2025.100297

**Published:** 2025-05-27

**Authors:** Aya Tanaka, Filippos T. Filippidis, Marie Line El Asmar, Anna Reed, Andrew Morley-Smith, Vasiliki Gerovasili

**Affiliations:** a Department of Primary Care and Public Health, School of Public Health, Imperial College London, White City Campus, 90 Wood Lane, London W12 0BZ, UK; b Department of Cardiothoracic Transplantation Harefield Hospital, Guy’s and St Thomas’ NHS Foundation Trust, Hill End Road, Uxbridge UB9 6JH, UK; c Department of Infectious Diseases, Imperial College London, South Kensington Campus, London SW7 2AZ, UK; d National Heart and Lung Institute, Imperial College London (Guy Scadding Building, Cale Street), London SW3 6LY, UK

**Keywords:** Lung transplantation, Heart transplantation, Pregnancy, Immunosuppression, HLTR

## Abstract

Immunosuppression advances have enabled organ transplant recipients to consider parenthood, but pregnancy poses risks to maternal and fetal health. This systematic review examines pregnancy outcomes and immunosuppression management in cardiothoracic transplant recipients. We conducted a literature search of PubMed/Medline, Embase, and Maternity and Infant Care Database in December 2022. We identified 54 relevant studies and data from the Transplant Pregnancy Registry International, covering 404 pregnancies from 272 heart recipients (HTR) and 148 pregnancies from 121 lung recipients (LTR). Live births occurred in 74.3% of HTR and 65.5% of LTR pregnancies (22% preterm). Graft dysfunction developed in 11.5% (during) and 12.4% (after) of HTR pregnancies and 17.6% (during) and 18% (after) of LTR pregnancies. Other complications included hypertension (HTR: 36.9%, LTR: 58.8%), preeclampsia (HTR: 19.7%, LTR: 12.2%), and diabetes (HTR: 11%, LTR: 27%). Mortality was 17.4% for HTR and 26.5% for LTR. Half of HTR and two-thirds of LTR were on Tacrolimus. Common immunosuppression changes included discontinuation of Mycophenolate Mofetil, Azathioprine, or Sirolimus with corticosteroid dose adjustment. Despite high successful pregnancy rates, heart and lung transplant recipients may face substantial risks of graft dysfunction and maternal death post-pregnancy.

## Background

Cardiothoracic transplantation outcomes have improved in recent decades. The 1-year mortality rate decreased from 18.8% in 1996-2006 to 10.4% in 2007-2019 and the 30-day mortality decreased as well from 11.0% in 1996-2006 to just 3.1% in 2007-2019.^1^ Patients live full lives and as part of that may wish to pursue pregnancy. However, pregnancy in heart and lung transplant recipients (HLTR) increases the risks of hypertension,^2^, ^3^ pre-eclampsia, diabetes, infections, and graft dysfunction.^4^ Physiologic changes during pregnancy and fluctuating immunosuppression levels heighten rejection risks, potentially leading to life-threatening complications.^5^ Furthermore, all immunosuppressants cross the placental barrier, and although certain drugs are considered safer in pregnancy, others, such as Mycophenolate Mofetil (MMF), increase the risks of structural anomalies of the fetus.^3^ All immunosuppressants can result in immunologic modifications of the fetus.^3^

Pregnancy outcome reports largely focus on kidney or liver transplants.^2^ The American Society of Transplantation guidelines on pregnancy in solid organ transplant recipients (2005) and The International Society of Heart and Lung Transplantation’s 2020 statement provide limited HLTR specifics.^3^, ^6^, ^7^

Existing reports on HLTR consist primarily of observational studies of small cohorts or case reports or have reported results mixed with other solid organ transplant recipients.^8^ In 1991, the American National Transplantation in Pregnancy Registry, a voluntary patient registry, was set up in North America.^9^ It’s now open to registrations worldwide, and in 2016, it changed its name to the Transplant Pregnancy Registry International (TPRI). In 28 years, 3,270 pregnancies in 1,806 transplant recipients have been registered in the TPRI, making it the largest source of information of pregnancy outcomes in solid organ transplantation.^10^ A previous systematic review by Acuna et al^11^ synthesized published data on pregnancy outcomes of HLTR; however, it excluded case reports and small case series. Given the scarcity of relevant literature, the addition of these data could have added value. Moreover, it focused mainly on pregnancy and peripartum complications with limited information on immunosuppression changes and monitoring.

The aim of this study was to systematically review the literature reporting pregnancy and delivery outcomes in HLTR. We also assessed the data on immunosuppression levels, dosing regimens, and monitoring, as well as complications of the mother and the fetus.

## Methods

### Search strategy and screening

A literature search was conducted using PubMed, Embase, and Maternity and Infant Care Database in December 2022. Search terms are detailed in Supplementary Table S1. Citations were compiled in EndNote^12^ and uploaded to Covidence^13^ for screening and quality assessment.

Studies were screened using predefined inclusion/exclusion criteria (Supplementary Table S2). Two reviewers independently screened each study, with a third settling any disagreements. After the full-text screening process, relevant data were extracted, and quality assessment (QATSDD) was conducted (Supplementary Table S3–1).^14^

### Data synthesis

Patients were divided in the Heart Transplant group and the Lung Transplant group. Recipients of both heart/lung transplants are reported in the Lung Transplant group. In three publications, outcomes of heart/lung or lung recipients (LTR) were reported together with heart recipients (HTR). We grouped these with the heart group. Case series and retrospective cohort studies (CS/RCS) are presented separately to case reports (CR). Data from the TPRI 2020 report are also presented separately.^15^

### Definition of mortality

Maternal mortality refers to female deaths related to or aggravated by pregnancy or its management. Our definition encompasses all deaths, regardless of the specific time period outlined in the WHO definition (which includes deaths during pregnancy, childbirth, or within 42 days of pregnancy termination).^16^

## Results

**Figure 1:**
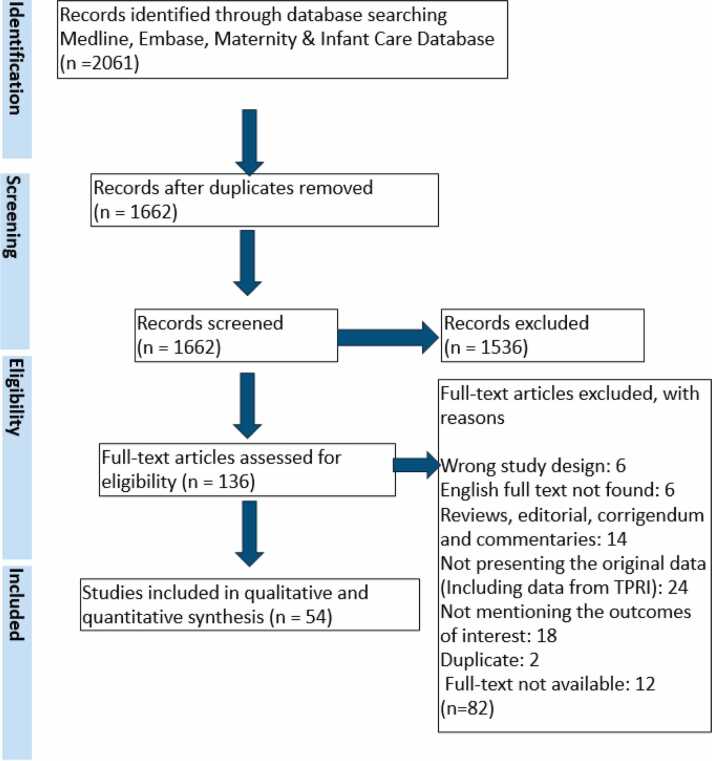
PRISMA flow diagram showing the selection process.

The search yielded 1662 unique publications. After reviewing 136 full-text articles, 54 were included (Table 1, Table 2) (Figure 1). Data were collected from 1995 to 2022 for heart and from 1996 to 2022 for lung transplants. There were 409 pregnancies in the heart group and 150 in the lung group (Table 3, Supplementary Table S4). US publications dominated the heart group (21.1%), while UK studies led in the lung group (35.3%) (Supplementary Table S5).

**Table 1:** The List of Citations and Characteristics of Patients and Pregnancies for *Heart* Transplant Recipients in Case Reports and Case Series/Retrospective Cohort Studies

Author, Year	Country, Setting	Study Period	Patients	Pregnancies	Transplant Indication	Age at Pregnancy (years)	Transplant-pregnancy interval (months)	Duration of maternal follow-up (months)	Study setting
*Case Reports*									
Lowenstein et al, 1988^17^	Argentina	1987	1	1	DCM	20	24	7	Sanatorio Guemes Hospital Privado
Camann et al, 1989^18^	US	1981-NR	1	1	PPCM	29	NR	0.5	Brigham and Women’s hospital
Hedon et al, 1990^19^	France	1987	1	2 (twin)	CHD	35	6	NR	Montpellier-Nimes Service
Liljestrand et al, 1993^20^	Sweden	1992	1	1	PPCM	30	27	18	Central Hospital, Karlskrona
Kurdi et al, 1994^21^	Saudi Arabia	1992	1	2	PPCM	25	9, 23	NR	Riyadh Armed Force Hospital
Ahner et al, 1994^22^	Australia	1992	1	1	DCM	23	4	1	Vienna University
Abukhalil et al, 1995^23^	UK	1995	1	1	MCM	19	36	NR	Harefield Hospital
Fleschler et al, 1995^24^	US	1994	1	1	NR	19	6	4 days	St. Luke’s Episcopal Hospital, Houston
Grimm et al, 1996^25^	Austria	1996	1	1	DCM	23	4	NR	University of Vienna
Dziatkowiak et al, 1996^26^	Poland	1992-1995	1	1	HCM	28	36	1.5	Collegium Medicum Jagiellonian University
Delforge et al, 1997^27^	US	1992-1995	1	2	CHD(A)	30	36	NR	Saint-Luc University Hospital, Brussels
Yuh-JerShen et al, 1997^28^	US	1996	1	1	NR	34	5	16	Kaiser Permanente Medical Center
Aramayo et al, 2000^29^	Brazil	1990-1997	1	1	DCM	20	72	5	Instituto de Cardiologia/IC-FUC
Barker et al, 2003^30^	UK	2003	1	1	MCM	20	96	1.5	Manchester Royal Infirmary
Ruygrok et al, 2004^31^	New Zealand	2003	1	1	DCM	33	54	6	Green Lane Hospital, National Women’s Hospital
Ginwalla et al, 2013^32^	US	NR	1	1	Other CM	27	204	5	NR
Kalinka et al, 2014^33^	Poland	2010	1	3	HCM	30	72, 96	NR	The Clinic of Perinatology of the Medical University of Lodz
Stribling et al, 2015^34^	US	2015	1	2	PPCM	23	36,42	12	St Thomas Medical Center, Nashville
Nitta et al, 2016^35^	Japan	2016	1	2	DCM	23	72, 75	1	The University of Tokyo
Liu et al,2018^36^	US	2018	1	1	DCM	24	264	NR	Loma Linda University Children’s Hospital
Couto et al, 2019^37^	Portugal	2006-2016	1	1	DCM	35	120	NR	Centro Hospitalar e Universitário de Coimbra
**Case series/Retrospective cohort studies**									
Scott et al, 1993^38^	US	NR	2	2	CHD:2	30, 36	48, 60	12, 8	University of Utah Medical Center
Haugen et al, 1998*^39^	Norway	NR	2	2	DCM:2	19,35	12,24	NR	NR
Tardivo, et al, 2004^40^	Italy	1991-2002	7	10	NR	29.6 (21-37)	83 (49-145)	NR	Heart transplant centers in Italy
Wasywich, et al, 2013^41^	New Zealand	1987-2010	2	3	NR:2	27 (Mean)	61,71,101	146.4 (Max)	New Zealand Heart Transplant Program
Bhagra et al, 2016^42^	UK	1985-2014	17	22	DCM:10 CHD:7	25 ± 5.8	98 ± 62.4	57 (Max)	Freeman Hospital, Newcastle upon Tyne, UK
Tsao et al, 2016^43^	Taiwan	2015	2	2	DCM:1 CHD:1	34,37	60,84	168, 24	National Taiwan University Hospital
Dagher et al, 2017^44^	Canada	1998-2016	8	18	CHD:4 CM:4	25.5(17.6-33.3)	120(31.2-324)	55.2 (14.4-206.4)	Four centers in Quebec
Sara Nunes et al, 2018^45^	Portugal	2007-2017	2	2	MCM:2	32, 28	72, 60	**NR**	Obscare and Clinico databases
D'Souza, et al, 2018^46^	Belgium, Canada	2001-2017	16	17	CM:10 CHD:6	28 ± 5.8	87.6 ± 48	67.2 (10-180)	University of Toronto, University Hospitals Leuven
Macera et al, 2018^47^	Italy	1985-2016	11	17	CHD:7 HCM:1 DCM:2 Other (CAD):1	33(23-36)	67(11-106)	108 (Mean)	Niguarda Great Metropolitan Hospital, Milan
LenkaVojtickova et al, 2019^48^	Czechia	NR	4	4	DCM:3 HCM:1	31 ± 2.0	67 ± 33	NR	Institut klinické a experimentální medicíny
Boyle et al, 2021^49^	Australia	2014-2018	3	5	Other CM:2 MCM:1	27 (23-38)	60(24-168)	NR	A single center in Queensland
Bedin et al, 2022^50^	France	2010-2018	9	11	NR	27.6 + −5.59	38.4 ± 9.1	N	Foch Hospital
Kuczaj et al, 2022^51^	Poland	1985-2021	8	8	DCM:3, HCM: 4, Other CM (Restrictive CM):1	25(19-35)	76.8(36-132)	73.2 ± 60	Silesian Center for Heart Diseases, Zabrze
Heart + Heart/lung + Lung (Included in the Heart group**): Retrospective cohort studies									
Troche et al 1998^52^	France	1984 -1996	H:6 HL:3	H:7 HL:3	H: CHD:2 HCM:3 HL: CHD:1 PPH:2	H31 (23-36)HL28 (24-31)	H22 (10-39)HL25 (11-34)	120 (Mean)	36 centers in France
Estensen et al, 2011^53^	Norway	NR	HL;6 H:19	42	CM:15 CHD: 9	26.5 (Mean)	**78** ± **38.4 (12-144)**	NR	Nordic Thoracic Transplant Centers
Mohamed-Ahmed et al, 2014^54^	UK	2007-2012	H:10 L:3 HL:1	14	CF:2 OLD:1 CM:4 DCM:2 CHD and PH:5	26 (20-38)	96(24-192)	NR	UK Obstetric Surveillance System (UKOSS)

*Included both the heart and lung group, because it included heart and heart/lung transplant recipients, whose outcomes can be clearly separated into two groups.

**Three studies^52^, ^53^, ^54^ were included in the heart group reported heart/lung or lung transplant outcomes together with heart transplant outcomes.

(A) single ventricle, great vessels transposition associated with a pulmonary artery stenosis

CF, Cystic fibrosis; CHD, Congenital heart disease; CM, Cardiomyopathy; DCM, dilated cardiomyopathy; H, Heart transplant recipients; HCM, Hypertrophic cardiomyopathy; HL, Heart and Lung transplant recipients; ILD, Interstitial lung disease; L, Lung transplant recipients; MCM, Myocarditis related cardiomyopathy; NR, Not reported; CAD, Coronary Artery Disease; OLD, Obstructive Lung Disease; PH, Pulmonary Hypertension; PPCM, Post/Peripartum cardiomyopathy.

**Table 2:** The List of Citations and Characteristics of Patients and Pregnancies for Lung Transplant Recipients in CR and CS/RCS

Author, Year	Country, Setting	Study period	Patients	Pregnancies	Transplant Indication		Age at pregnancy (years)	Transplant-pregnancy interval (months)	Duration of maternal follow-up (months)	Study setting
*Case Reports*										
Brackley et al, 1996^55^	UK	1996	1	1	CF	27		60	3	Queen's Medical Centre, Nottingham
Paradowski et al, 1996^56^	US	1995	1	1	OLD	35		24	10	University of North Carolina,
Parry et al, 1996^57^	UK	1988-1995	1	1	ILD	30		72	NR	Harefield Hospital.
Larciprete et al, 1999^58^	Italy	1998	HL:1	HL:1	PH:1	21		48	12	Ospedale Fatebenefratelli Isola Tiberina, Rome
Jongen et al, 2000^59^	Netherlands	2000	1	1	PH	36		48	6	University-based center in Netherlands
Kruszka et al, 2002^60^	UK	2002	1	1	ILD	32		48	NR	Naval Medical Center Portsmouth
Huang et al, 2010^61^	US	2010	1	1	CF	26		48	NR	Stanford Fertility and Reproductive Medicine Center
Divithotawela et al., 2015^62^	Australia	2012	1	2	CF	42		240	24	The Prince Charles Hospital
*Case series/Retrospective cohort studies*										
Parry et al, 1997^63^	UK	1985-1995	L:1 HL:3	L:1 HL:4	HL:PH:1 CHD:1 CF;1 L: CF:1	HL22/23,26,28 L28		HL26/40,25,32 L18	NR	Harefield hospital
Haugen et al, 1998*^39^	Norway	NR	HL:3	HL:3	CHD:3	22,39,24		72,168,48	36,24, NR	The National Hospital, University of Oslo
Rigg et al, 2000^64^	UK	1994-1996	HL:2	HL:3	OLD:1 CF:1	36,38,37		60,84,24	0.25	Royal Victoria Infirmary, Newcastle
Baron 2002^65^	France	1989-2000	HL:4	HL:5	CF:2 CHD:1 PPH:1	26. ± 22.3		37 ± 21 (6-64)	60±24 (36–96)	The thoracic Transplantation Unit of Nantes Hospital
Gyi et al,2006^66^	UK	1985-2003	HL:3 L:1	HL:3 L:1	CF:4	28, 30, 28, 22		30, 57, 33, 39	25,78,38, NR	The Royal Brompton and Harefield transplant center
Zurbano et al, 2012^67^	Spain	2010	L:1 HL:1	L:1 HL:1	L: ILD:1 HL:PH:1	30,38		72,7	NR	Hospital Marqués de Valdecilla, Hospital Universitario Puerta de Hierro
Thakrar et al, 2014^68^	Canada	NR	L:7 HL:7	19	CF:8 CHD:3 OLD:2 PH:1	31.4(22-39)		L 72(Mean) HL 86(Mean) (26-139)	NR	Freeman Hospital in Newcastle upon Tyne
Greene, et al, 2014^69^	US	NR	HL:4	5	CF:4	24 (21-29)		48 (12-96)	216 (204-228)	Keck School of medicine of the University of Southern California
Bry et al, 2019^70^	France	1991-2013	L:22 HL:13	39	CF:25 PH:7 OLD:3	28 ± 5		63 ± 44	55 ± 78	Nine transplantation centers in France

*Included both the heart and lung group.

CAD, Coronary Artery Disease; CF, Cystic fibrosis; CHD, Congenital heart disease; CM, Cardiomyopathy; DCM, dilated cardiomyopathy; H, Heart transplant recipients; HCM, Hypertrophic cardiomyopathy; HL, Heart &Lung transplant recipients; ILD, Interstitial lung disease; L, Lung transplant recipients; MCM, Myocarditis related cardiomyopathy; NR, Not reported; OLD, Obstructive Lung Disease; PH, Pulmonary Hypertension; PPCM, Post/Peripartum cardiomyopathy.

**Table 3:** Characteristics of HLTR and Pregnancy Details Reported in Case Reports (CR), Case Series and Retrospective Cohort Studies (CS/RCS), and the Transplant Registry International (TRPI)

	Heart			Lung		
	CS/RCS and CR	TPRI	Total	CS/RCS and CR	TPRI	Total
Total studies (*N*)	38	NR	38	17	NR	17
Total pregnancies (*N*)	217	187	404	94	54	148
Total recipients (*N*)	162	110	272	80	41	121
Mean maternal age (year) ***	28.1(38studies)	21	24.9	29.5(17studies)	26	28.2
Mean interval from transplantation to pregnancy (year)****	6.3(37studies)*	6.1	6.2	6.5(16studies)**	6.5	6.5
Mean follow-up period after pregnancy (year)****	5.8(20studies)	7.7	6.7	3.7(11studies)	4.2	3.9

* except for^61^

**except for^30^

*** Several publications reported the maternal age, interval from transplantation to pregnancy, and follow-up after pregnancy for each individual. In publications where only the mean was reported, we considered that the reported mean applied to each individual. Based on the above, mean age and follow up was calculated across all included studies.

**** The mean interval and follow-up period is also calculated the same as for calculating the average age.

NR, Not reported.

### Immunosuppression

**Figure 2:**
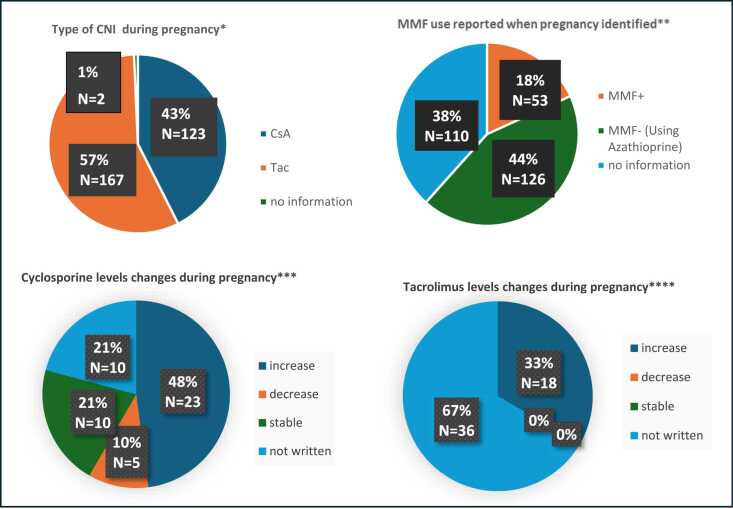
Immunosuppressant use and changes in pregnancy for the Heart group in CR/CS/RCS (28 studies) and TPRI**.***Footnote*: all numbers have been rounded to the closest integer in the pie charts above. * CsA: *N* = 123, 42.6% (CR/CS/RCS: 47.1%, TPRI: 40.1%), Tac: Total: *N* = 164, 56.7% (CR/CS/RCS:52.9%, TPRI: 58.8%), no information: Total *N* = 2, 0.7% (CR/CS/RCS: 0%, TPRI: 1.1%). **MMF+: Total *N* = 53, 18.3% (CR/CS/RCS: 15.6%, TPRI: 19.8%), MMF-(Using Azathioprine): Total *N* = 126, 43.6% (CR/CS/RCS: 39.2%, TPRI: 46.0%), no information: Total *N* = 110, 38.1% (CR/CS/RCS: 44.2%, TPRI: 34.2%). ***increase: *N* = 23, 47.9%, decrease: *N* = 5, 10.4%, stable: *N* = 10, 20.8%, not written: *N* = 10, 20.8% (These data come from CR/CS/RCS. TPRI does not report the changes in dose.). **** increase: *N* = 18, 33.3%, decrease: *N* = 0 0%, stable: *N* = 0, 0%, not written: *N* = 36, 66.7% (These data come from CR/CS/RCS. TPRI does not report the changes in dose.).

**Figure 3:**
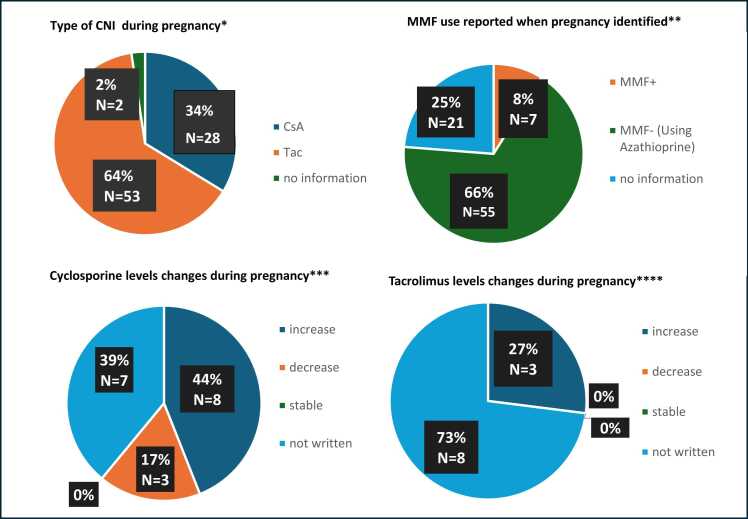
Immunosuppressant use and changes in pregnancy for Lung group in CR/CS/RCS (13 studies) and TPRI. All these numbers below have been rounded to the closest integer in the pie charts above: * CsA: Total *N* = 28, 33.7% (CR/CS/RCS: 62.1%, TPRI: 18.5%), Tac: Total *N* = 53, 63.9% (CR/CS/RCS: 37.9%, TPRI: 77.8%), no information: Total *N* = 2, 2.4% (CR/CS/RCS: 0%, TPRI: 3.7%). **MMF+: Total *N* = 7, 8.4% (CR/CS/RCS: 3.4%, TPRI: 11.1%), MMF-(Using Azathioprine): Total *N* = 55, 66.3% (CR/CS/RCS: 58.6%, TPRI: 70.4%), no information: Total *N* = 21, 25.3% (CR/CS/RCS: 37.9%, TPRI: 18.5%). ***increase: *N* = 8, 44.4%, decrease: *N* = 3, 16.7%, stable: *N* = 0, 0%, not written: *N* = 7, 38.9% (These data come from CR/CS/RCS. TPRI does not report the changes in dose.). **** increase: *N* = 3, 27.3%, decrease: *N* = 0, 0%, stable: *N* = 0, 0%, not written: *N* = 8, 72.7% (These data come from CR/CS/RCS. TPRI does not report the changes in dose.).

Figures 2 and 3, Table 4 demonstrate the immunosuppressant use and changes in dose/regimen in pregnancy for the HLTR, respectively.

**Table 4:** Immunosuppressant Use and Changes in Dose/Regimen in Pregnancy for Heart/Lung Group in CR/CS/RCS(28 Studies, 13 Studies, Respectively) and TPRI

Heart					Lung			
	CS/RCS and CR, *N* (%)		TPRI, *N* (%)		CS/RCS and CR, *N* (%)		TPRI, *N* (%)	
CNIs	CsA	Tac	CsA	Tac	CsA	Tac	CsA	Tac
	48/102(0.47)	54/102(0.53)	75/187 (0.40)	110/187 (0.59)	18/29 (0.62)	11/29 (0.38)	10/54 (0.19)	42/54 (0.78)
Increase	23*/48(0.48)	18**/54(0.33)	NR	NR	8*/18(0.44)	3**/11(0.27)	NR	NR
	Change in dose(mg)				Change in dose(mg)			
	450→600(46) 75→125(51) 250→300(47) 14/kg→20/kg(30) 14/kg→20/kg(33) 380→440(28) 250→300(36)	3.4→5.7(43) 12→20(41)			225→275:1(68)	4→5:2(76)7→9:1(76)		
Decrease	5***/48(0.10)	0/54	NR	NR	3***/18(0.17)	0/11	NR	NR
	Change in dose(mg)				Change in dose(mg)			
	240→200:1(47)	NR			400→300:1(72) 500→225:1(72) 300→225:1(72)	NR		
Change in regimen	CsA+Cor+Aza→CsA:1(34) CsA+Aza→CsA+Cor:2(46,55) CsA+Eve+Cor→CsA+Eve:1(51) CsA+Aza+Cor→CsA+Cor:4(55) CsA+Eve+Cor→CsA+Cor:1(55) CsA+Cor→CsA:1(55) CsA+Eve→CsA+Cor:1(55)	Tac+MMF→Tac+Cor:1(43) Tac+MMF→Tac+Aza:1(42) Tac+Sir→Tac+Aza:1(57) Tac+MPA→Tac+Aza:2(51) Tac+MPA+Cor→Tac+Aza+Cor:2(51) Tac+MMF+Cor→Tac+Cor:2(54) Tac+MMF+Sir→Tac:1(54) Tac+Aza+Cor→Tac+Cor:1(54) Tac+MPA→Tac:2(59)	NR	NR	NR	Tac+Aza +Cor→Tac+Cor:1(68) Tac+MMF+Cor→Cys+Aza+Cor:1(74)	NR	NR
Antimetabolites	Azathioprine	MMF	Azathioprine	MMF	Azathioprine	MMF	Azathioprine	MMF
	40/102 (0.39)	16/102 (0.16)	86/187 (0.46)	37/187 (0.20)	17/29(0.59)	1/29(0.03)	38/54 (0.70)	6/54 (0.11)
*Corticosteroids*	61/102 (0.60)		NR		20/29(0.69)		NR	
*Sirolimus*	2/102 (0.02)		17/187(0.09)		0/29		3/54(0.06)	

Aza, Azathioprine; CNIs, Calcineurin inhibitors; Cor, Corticosteroids; CsA, Cyclosporin; Eve, Everolimus; MMF, Mycophenolate mofetil; NR, Not reported; Sir, Sirolimus; Tac, Tacrolimus.

**<References>**

17, 18, 19, 20, 21, 22, 23, 24, 25, 26, 28, 29, 31, 32, 33, 34, 35, 38, 39, 40, 43, 44, 46, 47

48, 49, 51, 52

*^20^, ^22^, ^25^, ^26^, ^28^, ^29^, ^31^, ^38^, ^39^, ^42^, ^43^, ^47^, ^51^, ^52^

**^33^, ^34^, ^35^, ^43^, ^47^, ^49^, ^51^

***^39^, ^47^, ^52^

*** Methotrexate was part of the immunosuppression regimen in one unplanned pregnancy, which resulted in termination because of concerns about teratogenicity.

**<References>**

39, 55, 56, 58, 60, 62, 63, 64, 65, 66, 68, 69, 70

*^39^, ^56^, ^65^, ^66^

**^62^, ^66^, ^68^

***^64^

#### Heart transplant recipients

Twenty-eight publications and the TPRI report on immunosuppression use and changes during pregnancy. Of 289 HTR reporting information on calcineurin inhibitors (CNIs), 42.6% were on Cyclosporine (CsA), 56.7% on Tacrolimus. Furthermore, 6.6% were on Sirolimus, 43.6% on Azathioprine, and 18.3% on MMF. Among the 53 HTR who were on MMF, 26 (49.1%) resulted in a miscarriage and 27 (50.9%) resulted in livebirths, despite MMF being discontinued or switched to alternative medication upon confirmation of pregnancy. There was no information regarding adverse outcomes associated with azathioprine. Regarding Corticosteroid use, 61 HTR out of 102 (59.8%) reported using Corticosteroids in CR/CS/RCS; corticosteroid use is not reported in the TPRI.

Among 48 HTR on CsA in CR/CS/RCS, 11 (22.9%) reported a change in the immunosuppression regimen during pregnancy. Azathioprine was discontinued in seven pregnancies, Corticosteroids in three, and Everolimus in two. In three pregnancies Azathioprine or/and Everolimus were substituted by Corticosteroids. Among HTR on Tacrolimus in CR/CS/RCS, 13 out of 54 (24.1%) reported a change in the immunosuppression regimen during pregnancy. Azathioprine was discontinued in one HTR, and Sirolimus was substituted with Azathioprine in one HTR. MMF was discontinued in 11 HTR (substituted with Corticosteroids in one case and with Azathioprine in five, not substituted with anything in five, with three HTR remaining on Tacrolimus monotherapy).

Regarding changes in CsA dose during pregnancy in HTR reported in CR/CS/RCS, 23 out of 48 (47.9%) had a reported increase in the dose, five (10.4%) a reduction, and ten (20.8%) no change in dose. There is no information available on changes in CsA levels in the remaining ten pregnancies (20.8%). Regarding changes in Tacrolimus dose during pregnancy in HTR reported in CR/CS/RCS, 18 out of 54 (33.3%) had a reported increase in the dose of Tacrolimus during pregnancy. There is no information available on changes in Tacrolimus dose in the other 36 pregnancies (66.7%).

#### Lung transplant recipients

Thirteen publications and TPRI contained information about immunosuppression at the time of pregnancy. Among 83 LTR, 28 (18 in CR/CS/RCS, 10 in TPRI) (33.7%) were on CsA and 53 (11 in CR/CS/RCS, 42 in TPRI) (63.9%) were on Tacrolimus. In terms of antimetabolite use (Azathioprine/MMF), 55 (66.3%) were on Azathioprine and seven out of 83 (8.4%) were on MMF with no information on the use of antimetabolites in 21 recipients (25.3%). Among the seven LTR with exposure on MMF, the outcomes were three livebirths (42.9%), two miscarriages (28.6%), and one termination (14.3%). There was one neonatal death due to prematurity (26 weeks, umbilical cord anomaly).

Twenty out of 29 LTR (69.0%) reported using Corticosteroids. Corticosteroid use is not reported in the TPRI. Three pregnancies in LTR out of 54 (5.6%) were reported to be on Sirolimus in the TPRI. There is no Sirolimus use reported in CR/CS/RCS. Two regimen changes were reported in the Tacrolimus group (one discontinuation of Azathioprine use and one substitution of MMF with Azathioprine).

Regarding changes in CsA dose during pregnancy in LTR, 8 out of 18 (44.4%) had a reported increase and three (16.7%) a decrease in dose. Three out of 11 (27.3%) LTR receiving Tacrolimus had a reported increase in the dose during pregnancy with no cases reporting a dose reduction.

### Maternal deaths

Maternal deaths and outcomes are summarized in Table 5. We reported overall mortality rather than the WHO definition. We provide a breakdown of mortality during pregnancy and within the first year post-delivery instead.

**Table 5:** Maternal Outcomes in Heart/Lung Transplant Recipients Reported in Case Reports, Case Series/Case Cohorts and the TPRI Registry

Heart							
	CR		CS/RCS		TPRI (07/1987-04/2020)	Total (CR+CS/RCS+TPRI)	
	Studies = 21 Recipients = 21 Pregnancies = 28		Studies = 17* Recipients = 141 Pregnancies = 189		Recipients = 110 Pregnancies = 187	Studies = 38* Recipients = 272 Pregnancies = 404	
	Studies *N*(References)	Events (*N*/Pregnancies)	Studies *N*(References)	Events (*N*/Pregnancies)	Event	Events	
					(*N*/Pregnancies)	(%)	(*N*/Pregnancies)
Maternal death after delivery	0	0/0	7^41^, ^42^, ^46^, ^47^, ^51^, ^53^, ^54^	19/123	35/187	17.4	54/310^&^
Graft rejection/Drop in LVEF*** during pregnancy	5^25^, ^27^, ^28^, ^30^, ^35^	5/5	6 (38, 41, 42, 46, 52, 54)^&, &^	9/68	15/187	11.5	29/260
Graft rejection/Drop in LVEF***/Graft vasculopathy after delivery	2^32^, ^34^	1/2	6^44^, ^47^, ^48^, ^51^, ^53^, ^54^	12/103	NR	12.4	13/105
Hypertensive disorders of pregnancy	3^19^, ^24^, ^37^	3/3	10^38^, ^42^, ^44^, ^45^, ^46^, ^48^, ^51^, ^52^, ^53^, ^54^	29/141	99/187**	36.9	124/331
Pre-eclampsia	1^31^	1/1	8^38^, ^43^, ^44^, ^45^, ^46^, ^49^, ^53^, ^54^	11/131	51/187	19.7	63/319
Diabetes treated	0	0/0	5^42^, ^44^, ^46^, ^53^, ^54^	18/113**	15/187**	11.0	33/300
Cesarean deliveries	16 Except for^20^, ^22^, ^23^, ^26^, ^32^	5/18 live births	15 (Except for^48^, ^49^)	78/140 live births	59/131 live births	49.1	142/289 livebirths
*Lung*							
	CR		CS/RCS		TPRI (06/1992-03/2020)	Total (CR+ CS/RCS+TPRI)	
	Studies = 8 Recipients = 8 Pregnancies = 9		Studies = 9* Recipients = 72 Pregnancies = 85		Recipients = 41 Pregnancies = 54	Studies = 17* Recipients = 121 Pregnancies = 150	
	Studies *N*(References)	Events (*N*/Pregnancies)	Studies *N*(References)	Events (*N*/Pregnancies)	Events (*N*/Pregnancies)	Events	
						(%)	(*N*/Pregnancies)
Maternal death before delivery	8	0/9	1^70^	1/39	NR	2.1	1/48^&,&,&^
Maternal death after delivery	0	0/0	4^63^, ^66^, ^68^, ^70^	20/63	11/54	26.5	31/117^&,&,&,&^
Graft rejection/Drop in FEV1^****^ during pregnancy	4^56^, ^57^, ^60^, ^62^	4/6	4^39^, ^63^, ^66^, ^68^	5/31	7/54	17.6	16/91
Graft rejection/Drop in FEV1**** after delivery	2^57^, ^60^	2/2	5^39^, ^63^, ^66^, ^68^, ^70^	18/70	3/54	18.3	23/126
Hypertensive disorders of pregnancy	2^57^, ^62^	2/3	4^64^, ^66^, ^69^, ^70^	6/12	32/54**	58.0	40/69
Pre-eclampsia	1^62^	1/1	4^63^, ^68^, ^69^, ^70^	8/68	6/54	12.2	15/123
Diabetes treated	0	0/0	4^63^, ^68^, ^69^, ^70^	17/68**	16/54**	27.0	33/122
Cesarean deliveries	6^55^, ^56^, ^57^, ^60^, ^61^, ^62^	3/7 live births	7 (Except for^65^)	16/51 live births	16/35 live births	37.6	35/93 live births

*: 1 paper by Haugen 1998^39^ is included both in the heart and lung group.

**: Both underlying +occurring first time in pregnancy.

***: Left ventricular ejection fraction.

****: Forced expiratory volume.

&: The denominator is the number of pregnancies (404) for which data are available (310). Some recipients will have had multiple pregnancies.

&,&: Excluding one patient (59) with a mild drop in LVEF in whom rejection was excluded.

&,&,&: Te denominator is the number of pregnancies (148) for which data are available (48). Some recipients will have had multiple pregnancies.

&,&,&,&: The denominator is the number of pregnancies (148) for which data are available (117) Some recipients will have had multiple pregnancies.

#### Heart transplant recipients

There were 54/310 (17.4%) cases of maternal deaths after pregnancy in HTR reported in 7 CR/CS/RCS (19/123) and TPRI (35/187). In 31 studies, maternal outcome was not reported. Among the 19 deaths from 123 HTR reported in CR/CS/RCS, three HTR died within 1 year after delivery. Of those, one HTR died immediately after delivery due to postpartum hemorrhage and two HTR died 10 and 11 months after delivery, respectively, due to heart failure. Two deaths did not include information on the timing of the death. The remaining 14 heart transplant recipients (HTR) (73.7%) died more than 1 year after delivery. The causes of death after the first year post-delivery were acute rejection (two cases), heart failure (one case), lymphoma (one case), bronchiolitis obliterans (two cases), graft rejection believed to be secondary to non-adherence to immunosuppressant medications (four cases), hepatic cirrhosis during concomitant hepatitis (one case), and allograft vasculopathy (two cases). The cause of death was not reported in three HTR. TPRI does not report the cause nor the timing of maternal deaths. The mean age of children at the time of maternal death was 3.6 years (after delivery-14 years) for CR/CS/RCS and 10 years for TPRI.

#### Lung transplant recipients

Thirty-one out of 117 (26.5%) women in 4 CR/CS/RCS (20/63) and TPRI data (11/54) died after pregnancy during the average 6.5 years of follow-up for TPRI and 3.7 years (0.25-16.4 years) for CR/CS/RCS. One patient died during pregnancy (six weeks after conception) due to acute pneumonitis. There were reports of three deaths within the first year after delivery (reported in CR/CS/RCS). Five deaths do not include information of the timing of the death. The remaining 11 LTR (55.0%) died more than one year after delivery. The causes of death were chronic allograft dysfunction (CLAD) (nine cases), pulmonary infection (one case), pulmonary embolism (one case) and heart failure (one case). The cause of death was not mentioned in eight recipients. TPRI does not report the cause nor the timing of maternal deaths.

### Graft dysfunction during and after pregnancy

#### Heart transplant recipients

Graft dysfunction (including episodes of acute rejection, a drop in Left Ventricular Ejection Fraction (LVEF) not attributed to other causes, and graft vasculopathy) during and after pregnancy is reported in Table 5. Twenty nine out of 260 (11.5%) HTR developed graft dysfunction during pregnancy, and 13 out of 105 (12.4%) after delivery. Of 38 CR/CS/RCS, 47.4% included baseline LVEF data.

#### Lung transplant recipients

Sixteen out of 91 (17.6%) LTR developed graft dysfunction (including episodes of acute rejection, development of CLAD, and/or irreversible drop in FEV1 with exclusion of infective and other etiology) during pregnancy, and 23 out of 126 (18.3%) after delivery (Table 5). Among all 17 studies reviewed, nine CR/CS/RCS (52.9%) included baseline FEV1 data.

### Hypertensive disorders of pregnancy, pre-eclampsia and diabetes

#### Heart transplant recipients

Hypertensive disorder during pregnancy was reported in 122 of 331 HTR (36.9%) (13 CR/CS/RCS and TPRI), pre-eclampsia in 63 of 319 HTR (19.7%) (9 CR/CS/RCS and TPRI), and diabetes during pregnancy in 33 of 300 HTR (11.0%) (five CR/CS/RCS and TPRI).

#### Lung transplant recipients

Hypertensive disorder was reported in 40 of 69 LTR (58.0%) (6 CR/CS/RCS and TPRI), pre-eclampsia in 15 of 123 LTR (12.2%) (5 CR/CS/RCS and TPRI), and diabetes during pregnancy in 33 of 122 LTR (27%) (4 CR/CS/RCS and TPRI). However, these figures refer to women undergoing treatment for hypertensive disorders and diabetes during pregnancy; hence, they are not limited to conditions diagnosed during pregnancy for the first time.

### Cesarean deliveries

#### Heart transplant recipients

There were 142 cesarean sections out of 289 (49.1%) livebirths (31 CR/CS/RCS and TPRI).

#### Lung transplant recipients

There were 35 cesarean sections out of 93 (37.6%) livebirths (13 CR/CS/RCS and TPRI).

### Fetal outcomes

#### Heart transplant recipients

Three hundred pregnancies out of 404 (74.3%) (37 CR/CS/RCS and TPRI) led to live birth. It was not possible to determine the outcome of 17 pregnancies. There were four neonatal deaths and 18 congenital anomalies among 283 livebirths (33 CR/CS/RCS and TPRI). Among the 53 MMF exposures recognized during pregnancy, MMF was stopped or switched to alternative medication upon confirmation of pregnancy. However, 26 of those pregnancies in HTR (49.1%) resulted in a miscarriage, and the remaining 27 in livebirths. Among livebirths, 15.0% had birth defects. Birth defects included duodenal atresia, AV canal defect, tetralogy of Fallot; facial deformities and laryngomalacia (one case each). Among 236 pregnancies without MMF exposure, birth defects included: cystic hygroma, vermian hypoplasia of the cerebellum, hypospadias, undescended testicle, pectus excavatum, hydronephrosis, and tongue-tie (Table 6).

**Table 6:** Fetal Outcomes in Heart/Lung Transplant Recipients Reported in Case Reports, Case Series/Case Cohorts and the TPRI Registry

Heart						
	CR		CS/RCS		TPRI (07/1987-04/2020)	Total (CR + CS/RCS + TPRI)
	Studies = 21 Recipients = 21 Pregnancies = 28		Studies = 17* Recipients = 141 Pregnancies = 189		Recipients = 110 Pregnancies = 187	Studies = 38* Recipients = 272 Pregnancies = 404
	Studies (*N*)	Events (*N*/Pregnancies)	Studies (*N*)	Events (*N*/Pregnancies)	Events	Events
					(*N*/Pregnancies)	(*N*/Pregnancies)
Live births	20(a)	20/28	17	149/189	131/187	300/404
Pregnancy terminations	20(a)	3/28	17	13/189	8/187	24/404
Miscarriages	20(a)	4/28	17	25/189	52/187	81/404
Stillbirth	20(a)	0/28	17	0/189	2/187	2/404
Premature birth	20(a)	7/20 live births	13(b)	19/132 livebirths	48/131 live births	74/283 live births
Neonatal death	20(a)	0/20 live births	13(b)	4/132 live births	０/131 live births	4/283 live births
Congenital anomalies	20(a)	2/20 live births	13(b)	5/132 live births	11/131 live births	18/283 live births
*Lung*						
	CR		CS/RCS		TPRI (06/1992-03/2020)	Total (CR+CS/RCS+TPRI)
	Studies = 8 Recipients = 8 Pregnancies = 9		Studies = 9* Recipients = 72 Pregnancies = 85		Recipients = 41 Pregnancies = 54	Studies = 17* Recipients = 121 Pregnancies = 150
	Studies (*N*)	Event (*N*/Pregnancies)	Studies (*N*)	Event (*N*/Pregnancies)	Events	Events
					(*N*/Pregnancies)	(*N*/Pregnancies)
Live births	8	8/9	9	54/85	35/54	97/148
Pregnancy terminations	8	2/9	9	11/85	5/54	18/148
Miscarriages	8	0/9	9	14/85	15/54	29/148
Stillbirth	8	0/9	7(c)	0/85	0/54	0/148
Premature birth	8	4/8 live births	7(c)	7/46 live births	19/35 live births	30/89live births
Neonatal death	8	0/8 live births	7 (c)	1/46 live births	3/35 live births	4/89 live births
Congenital anomalies	8	1/8 live births	7 (c)	2/46 live births	3/35 live births	6/89 live births

*1 paper by Haugen 1998^39^ is included both in the heart and lung group.

**<References>** a. Except for,^32^ b. Except for,^41^, ^46^, ^49^, ^53^ c. Except.^64^, ^68^

#### Lung transplant recipients

Ninety-seven pregnancies out of 148 (65.5%) (17 CR/CS/RCS and TPRI) led to live birth. There were four neonatal deaths and six congenital anomalies among 89 livebirths (15 CR/CS/RCS and TPRI). The fetal outcome was not reported in eight pregnancies. Among the seven recipients using MMF during the first trimester, two pregnancies resulted in miscarriage, three resulted in live births (no birth defects), and there was one termination and neonatal death due to prematurity (26 weeks, umbilical cord anomaly) (Table 6).

### Risk of bias assessment

Most studies were rated highly in terms of having explicit aims and objectives, description of the research setting, and fit between stated research question and method of data collection (Supplementary table S3–2). However, most of the studies did not report an explicit theoretical framework, evidence of sample size consideration, rationale for choice of data collection tool, good justification for analytical method selected, assessment of reliability of analytical process, and evidence of user involvement in design. Some studies may have received a low score simply because a specific criterion was not described in detail in the manuscript, even though the study authors may have considered it.

## Discussion

This systematic review of 54 CR/CS/RCS and the TPRI database reports on pregnancy outcomes, complications, and immunosuppression management in HLTR. Due to scarce literature, we included CR and data from TPRI’s annual report.^10^ This resulted in the largest number of pregnancies in HLTR ever reported: 549 pregnancies in 390 HLTR, significantly more than a previous review that excluded CR and was based on an earlier TPRI report.^11^ Heart/lung transplant recipients are grouped with lung transplant recipients. In three studies the outcomes of 10 heart/lung transplant recipients were reported together with the heart transplant recipients’ outcomes and could not be separated for the analysis.

This is the first systematic review to synthesize available data on graft dysfunction after pregnancy in HLTR. We found a high incidence of graft dysfunction during and after pregnancy in both HTR and LTR. In HTR, 11.5% and 12.4% developed graft dysfunction during and after pregnancy (mean follow up period of 6.7 years), respectively. This is comparable to a previous systematic review which showed a 9.4% risk of graft rejection during pregnancy.^11^ New graft dysfunction may result from underlying graft insufficiency or new cardiac injury. The cardiac graft is required during pregnancy to handle additional blood volume and increase cardiac output, and the acute dysfunction during pregnancy may reflect pre-existing abnormalities or reduced cardiac reserve already present in the graft and decompensated by these increased demands. Equally there may be new immunological injury with acute rejection in the context of any of new allosensitisation, reduced immunosuppression, or low levels of CNI due to failure of levels monitoring. Any new graft dysfunction is difficult to diagnose and treat during the pregnancy.

More alarmingly in LTR, 17.6% and 18.3% developed graft dysfunction during and after pregnancy (mean follow up period of 3.9 years), respectively. The frequency of graft rejection during pregnancy is higher than what reported by Acuna et al (17.2% vs 9.8%).^11^ Higher percentages may reflect the inclusion of CR, likely focusing on recipients with complications. Indeed, if we exclude CR from this analysis, the percentages during pregnancy are 14.1%, which is closer to what has been reported in the previous review.^11^ With regards to 18.3% of LTR developing graft dysfunction after pregnancy, it is difficult to find a comparable cohort of LTR without a history of pregnancy; however, these finding should be interpreted in the context of 60% of LTR developing BOS by the time they are 5 years post-transplant.^71^ Finally, it is possible that allograft dysfunction observed during pregnancy may be related to processes that predate conception and therefore cannot be conclusively attributed to the pregnancy itself.

Maternal mortality rates after delivery for HTR and LTR in this systematic review are 17.1% and 26.5%, respectively. On the other hand, maternal mortality rates after delivery for kidney and liver transplant recipients, TPRI reported that 50% and 42% of deliveries were cesarean sections, are reported to be 18% and 13%, respectively.^10^ This is an important figure because physicians and recipients need to be aware of the risks when discussing issues around pregnancy. However, it is difficult to compare risks, considering that follow-up periods differ depending on the source. Our review showed a mean follow-up of 5.8 years for HTR and 3.7 years for LTR, while TPRI reported 7.7 years for HTR and 4.1 years for LTR. Such variation can make a big difference, considering that women of reproductive age who have received a heart or lung transplant are faced with substantial mortality risks; the 5-year survival post-lung and post-heart transplantation for this group is between 61.5% and 75%, and about 85%, respectively. Despite these limitations, our comprehensive review can provide useful data that can inform discussions about the implications of pregnancy for the patients, their partners, and children. While the WHO definition of maternal death pertains to the death of a woman during pregnancy or within 42 days of pregnancy termination, it is essential to extend the follow-up period to include a longer duration, considering the potential for deaths exacerbated by underlying conditions.

This is the first systematic review to synthesize the available information regarding immunosuppression management, including changes in immunosuppression regimens and levels during pregnancy in HLTR. In HTR, the most common changes in the immunosuppression regimen were discontinuation of MMF, followed by discontinuation of Azathioprine, Everolimus/Sirolimus and corticosteroids. Similarly, in lung transplant recipients the most common change was discontinuation of MMF, but most patients remained on Azathioprine. Although it is possible that immunosuppression strategies were tailored to patients’ requirements, these changes suggest CNIs (either Tacrolimus or Cyslosporine) as the cornerstone of immunosuppression during pregnancy with use of Azathioprine and Corticosteroids as clinically indicated, as well as the discontinuation of MMF and mTOR inhibitors which should not be used during pregnancy.^7^ As MMF is known to be teratogenic^72^ treatment with MMF at the time of pregnancy suggests that these pregnancies were unplanned; despite discontinuation of MMF, 49% of those pregnancies resulted in miscarriage, highlighting the importance of pre-transplant counseling with regards to the risks of both immunosuppression medications and pregnancy itself. Ongoing discussion in the post-transplant phase is desirable. Many women of child-bearing age have not had to consider their fertility in the context of severe, life-limiting heart/lung disease. Their prompt return to good health with transplant is accompanied by an unexpected return to normal fertility, and as such, clear messaging around this is vital. Multifaceted precounception councelling should include advice on contraception, risks of pregnancy, and the importance of appropriate pregnancy planning, which should include both parents as well a psychology input.^7^ A multidisciplinary team including—but not limited to- the transplant team, maternal-fetal medicine specialists, anaesthesiologists, transplant pharmacist and psychologist should be involved throughout pregnancy to ensure coordinated care for the best maternal, fetal, and neonatal outcomes.

Regarding CNI level changes during pregnancy, almost half of HTR and LTR required an increase in their CsA dose (47.9% and 44.4%, respectively) with the rest requiring a reduction or no change, whereas approximately one third of HTR and LTR recipients required an increase in their Tacrolimus dose (33.3% and 27.3%, respectively) with no reports of dose reduction. This is similar to other solid organ transplant recipients.^73^, ^74^ During pregnancy, reduced gastrointestinal motility, vomiting, increased plasma volume and fat stores, as well as changes in plasma-binding protein concentrations and drug metabolism can result in unpredictable changes of levels of CNI in the blood. Consequently, the International Society of Heart and Lung Transplantation consensus statement recommends more frequent therapeutic drug monitoring preferably every 2-4 weeks during pregnancy.^7^

Across all data sources in our review 36.9% of HTR and 58.0% of LTR required treatment for hypertensive disorders and 10.0% of HTR and 27.0% of LTR for diabetes. Hypertensive disorders may lead to Intrauterine Growth Restriction, Small for Gestational Age, placental abruption, and emergency cesarean section in case the condition becomes severe, like pre-eclampsia.^75^, ^76^ Large birth weight babies and obstructed labor are common complications of diabetes during pregnancy.^77^ In addition, people who develop hypertension or diabetes during pregnancy are more likely to develop chronic hypertension or diabetes mellitus in later life. Exposure to hyperglycemia in the womb predisposes children to a high risk of becoming overweight or obese and developing Type 2 diabetes, hypertension, and renal diseases.^75^, ^76^, ^77^

Comparatively, in the general population of pregnant women worldwide, the incidence of hypertensive disorders of pregnancy is approximately 2.73%, with chronic hypertension, preeclampsia, and eclampsia occurring at rates of 0.29%, 2.16%, and 0.28%, respectively.^78^ The pooled global standardized prevalence of Gestational Diabetes Mellitus is 14.0% (95% confidence interval: 13.97-14.04).^79^ When comparing these numbers with those of HLTR, it appears that HLTR are more susceptible to hypertensive disorders.

The percentage of cesarean section for HLTR was almost two-fold compared to the general population in the UK (about 25%)^80^ and nearly 1.5 times the rate in the general population in the US (about 34%).^81^ TPRI reported that 50% of kidney and 42% of liver transplant deliveries were cesarean sections, similar to HTR and LTR. This suggests that practice does not adhere to guidelines reserving cesarean sections for obstetric indications or acute heart failure.^82^ This may be due to the fact that clinicians are concerned about the physiological impact of vaginal delivery in heart or lung transplant recipients whereas they may feel they have better control by c-section or it may be influenced by the higher incidence of pregnancy complications or it could also be down to patient choice.

Preterm delivery was also more frequent in HLTR in our review (26.1% for HTR and 33.7% for LTR) compared to the general population in the UK (6.9%) and in the US (9.3%) in 2019.^80^, ^83^ The registry illustrated that the median gestation age was 32 and 34 weeks for LTR and HTR, respectively, meaning that most babies had low birth weight in this population.^10^ Possible reasons are both fetus and mother complications, including Small for Gestational Age, Intrauterine Growth Restriction, pre-eclampsia, and hypertension.^84^, ^85^

### Strengths and limitations

Our systematic review included small case series and CR because there have been considerably fewer publications on pregnancy after heart and lung transplantation compared with liver or kidney transplantation. This allowed us to use a larger dataset than previous reviews. However, CR may overestimate risks. Overall, available studies were of moderate quality, and interpretation of our findings should be made with caution. Data collected from TPRI may also be subject to bias, as contribution of data is voluntary, and it is likely that successful outcomes may be overrepresented. Although we excluded publications that summarized data from the TPRI, it is possible that some cases may be counted twice. Most of the previous studies on the topic simply report the regimen of immunosuppression, whereas we reported the changes both in immunosuppression regimens and in CNIs dose changes. We are also the first to summarize available data regarding graft dysfunction/rejection after pregnancy. Furthermore, there was wide variation in both definitions and follow-up periods across our data sources, which might influence the findings. For example, the definition of gestational diabetes and the diagnostic distinction between preeclampsia and hypertension were inconsistent across different studies. The included publications also report data across different geographic regions and for HLTR in various socioeconomic and healthcare contexts, which makes data synthesis challenging.

## Conclusion

While live births are possible among HLTR, there may be a high risk of new graft dysfunction and maternal death post-pregnancy. We described the frequency of obstetric complications and delivery outcomes. The high incidence of these complications underscores the need for preconception counseling and multidisciplinary care before, during, and after pregnancy. This review can inform preconception counseling and support during and after pregnancy. Future studies should consider a systematic assessment of pregnancy outcomes in cardiothoracic transplant recipients by interrogating national obstetric registries.

## Disclosure Statement

This research did not receive any specific grant from funding agencies in the public, commercial, or not-for-profit sectors.

## Declaration of Competing Interest

The authors declare the following financial interests/personal relationships which may be considered as potential competing interests: Vasiliki Gerovasili reports a relationship with Boehringer Ingelheim GmbH that includes: speaking and lecture fees. Vasiliki Gerovasili reports a relationship with Takeda Pharmaceutical Company Limited that includes: travel reimbursement. If there are other authors, they declare that they have no known competing financial interests or personal relationships that could have appeared to influence the work reported in this paper.

## Footnotes

Appendix A Supplementary data associated with this article can be found in the online version at doi:10.1016/j.jhlto.2025.100297.

## References

[1] Mancini D., Gibson G.T., Rangasamy S. (2021). Improving survival after heart transplantation despite increasing complexity. Eur Heart J.

[2] Armenti V.T., Constantinescu S., Moritz M.J., Davison J.M. (2008). Pregnancy after transplantation. Transpl Rev.

[3] Vos R., Ruttens D., Verleden S.E. (2014). Pregnancy after heart and lung transplantation.

[4] Thakrar M.V., Morley K., Lordan J.L. (2014). Pregnancy after lung and heart-lung transplantation. J Heart Lung Transpl Off Publ Int Soc Heart Transpl.

[5] Radomski J.S., Ahlswede B.A., Jarrell B.E. (1995). Outcomes of 500 pregnancies in 335 female kidney, liver, and heart transplant recipients. Transpl Proc.

[6] Shaner J., Coscia L.A., Constantinescu S. (2012). Pregnancy after lung transplant. Prog Transpl.

[7] Kittleson M.M., Defilippis E.M., Bhagra C.J. (2023). Reproductive health after thoracic transplantation: an ISHLT expert consensus statement. J Heart Lung Transpl.

[8] Punnoose L.R., Coscia L.A., Armenti D.P., Constantinescu S., Moritz M.J. (2020). Pregnancy outcomes in heart transplant recipients. J Heart Lung Transpl.

[9] Armenti V.T., Daller J.A., Constantinescu S. (2006). Report from the National Transplantation Pregnancy Registry: outcomes of pregnancy after transplantation. Clin Transpl.

[10] Transplant Pregnancy Registry International, 2020 Annual Report, Gift of Life Institute, Philadelphia, PA, USA.

[11] Acuna S., Zaffar N., Dong S., Ross H., D'Souza R. (2020). Pregnancy outcomes in women with cardiothoracic transplants: a systematic review and meta-analysis. J Heart Lung Transpl.

[12] Endnote. Available at: https://endnote.com.

[13] Covidence systematic review software, Veritas Health Innovation, Melbourne, Australia. Available at www.covidence.org.

[14] Sirriyeh R., Lawton R., Gardner P., Armitage G. (2012). Reviewing studies with diverse designs: the development and evaluation of a new tool. J Eval Clin Pract.

[15] Michael J. Moritz M. Transplant Pregnancy Registry International (Formerly National Transplantation Pregnancy Registry) 2020 Annual Report. In: Serban Constantinescu M, PhD, editor. 2021.

[16] Say L., Chou D., Gemmill A. (2014). Global causes of maternal death: a WHO systematic analysis. Lancet Glob Health.

[17] Lowenstein B.R., Vain N.W., Perrone S.V., Wright D.R., Boullon F.J., Favaloro R.G. (1988). Successful pregnancy and vaginal delivery after heart transplantation. Am J Obstet Gynecol.

[18] Camann W.R., Goldman G.A., Johnson M.D., Moore J., Greene M. (1989). Cesarean delivery in a patient with a transplanted heart. Anesthesiology.

[19] Hedon B., Montoya F., Cabrol A. (1990). Twin pregnancy and vaginal birth after heart transplantation. Lancet.

[20] Liljestrand J., Lindström B. (1993). Childbirth after post partum cardiac insufficiency treated with cardiac transplant. Acta Obstet Gynecol Scand.

[21] Kurdi A.M., Halim M.A., Nuaim M.A., El Torkey M.M. (1994). Successive pregnancies in a patient with a transplanted heart. J Obstet Gynaecol.

[22] Ahner R., Kiss H., Zuckermann A. (1994). Pregnancy and spontaneous delivery 13 months after heart transplantation. Acta Obstet Gynecol Scand.

[23] Abukhalil I.E.H., Govind A. (1995). Pregnancy in heart transplant recipients. Case report and review. Clin Exp Obstet Gynecol.

[24] Fleschler R.G., Sala D.J. (1995). Pregnancy after organ transplantation. J Obstet Gynecol Neonatal Nurs JOGNN/NAACOG.

[25] Grimm M., Ahner R., Simon P. (1996). Pregnancy and uncomplicated vaginal delivery only 13 months after cardiac transplantation (case report). Acta Chirurgica Austriaca.

[26] Dziatkowiak A., Zdebski Z., Tracz W. (1996). Successful full-term pregnancy in a patient three and a half years after a heart transplant. Ann Transpl Q Polish Transpl Soc.

[27] Delforge C., Kartheuser R., De Plaen J.F., Goenen M., Hubinont C. (1997). Pregnancy after cardiac transplantation. Transpl Proc.

[28] Yuh-Jer Shen A., Mansukhani P.W. (1997). Is pregnancy contraindicated after cardiac transplantation? A case report and literature review. Int J Cardiol.

[29] Aramayo A.M., Nunes E Silva D., Grundler C., Nesralla I., Bordignon S. (2000). Pregnancy after cardiac transplantation. Report of one case and review. Arquivos Brasileiros de Cardiologia.

[30] Barker T.A., Cotter L. (2003). Pregnancy following heart trasplantation: a case report. Br J Cardiol.

[31] Ruygrok P.N., Gibbs H., Coverdale H.A., Wilkinson L., Wasywich C.A. (2004). Planned pregnancy in a heart transplant recipient. Int Med J.

[32] Ginwalla M., Khush K.K., Pando M.J. (2013). Pregnancy-related human leukocyte antigen sensitization leading to cardiac allograft vasculopathy and graft failure in a heart transplant recipient: a case report. Transpl Proc.

[33] Kalinka J., Szubert M., Zdziennicki A. (2014). A second delivery after heart transplantation—a case study. Kardiochirurgia i Torakochirurgia Polska = Polish J Cardio-Thoracic Surg.

[34] Stribling W.K., Flattery M.P., Smallfield M.C., Kimball P., Shah K.B. (2015). Pregnancy-related allograft rejection following heart transplant. Prog Transpl.

[35] Nitta D., Kinugawa K., Imamura T. (2016). Successful pregnancy and delivery in a heart transplantation recipient. Int Heart J.

[36] Liu Y., Bock M.J., Gold J.A. (2018). The importance of preconception and prenatal genetic evaluation in heart transplant individuals and fetal and postnatal cardiac monitoring in their offspring. Cardiol Young.

[37] Couto D., Guerra N., Pais A.S., Sousa A.P., Almeida-Santos T. (2019). Fertility preservation with successful pregnancy outcome in a patient with transplanted heart and non-Hodgkin's lymphoma-a case report. BMC Pregnancy Childbirth.

[38] Scott J.R., Wagoner L.E., Olsen S.L., Taylor D.O., Renlund D.G. (1993). Pregnancy in heart transplant recipients: management and outcome. Obstet Gynecol.

[39] Haugen G., Aass H., Ihlen H. (1998). Pregnancy in heart and heart-lung transplant recipients. Acta Obstet Gynecol Scand.

[40] Tardivo I., Centofanti P., Goggi C. (2004). Pregnancy in heart transplant recipients. J Heart Lung Transpl.

[41] Wasywich C.A., Ruygrok A.M., Gibbs H., Painter L., Coverdale H.A., Ruygrok P.N. (2013). Exploring parenthood in the New Zealand Heart Transplant Program. Transpl Proc.

[42] Bhagra C.J., Bhagra S.K., Donado A. (2016). Pregnancy in cardiac transplant recipients. Clin Transpl.

[43] Tsao C.I., Chou N.K., Chi N.H. (2016). Surveillance of immunosuppression during pregnancy after heart transplantation: case report. Transpl Proc.

[44] Dagher O., Alami Laroussi N., Carrier M. (2018). Pregnancy after heart transplantation: A well-thought-out decision? The Quebec provincial experience—a multi-centre cohort study. Transpl Int Off J Eur Soc Organ Transpl.

[45] Sara Nunes S., Araujo P., Machado A.P., Montenegro N., Amorim S. (2018). Pregnancy after cardiac transplantation: maternal and fetal outcomes in a Portuguese tertiary hospital. Eur J Heart Failure.

[46] D'Souza R., Soete E., Silversides C.K. (2018). Pregnancy outcomes following cardiac transplantation. J Obstet Gynaecol Can.

[47] Macera F., Occhi L., Masciocco G., Varrenti M., Frigerio M. (2018). A new life: motherhood after heart transplantation. A single-center experience and review of literature. Transplantation.

[48] Lenka Vojtickova L., Kubanek M., Podzimkova M. (2019). Pregnancy in patients with pre-existing cardiomyopathy and in cardiac transplant patients. Eur J Heart Failure.

[49] Boyle S., Sung-Him Mew T., Lust K., McKenzie S., Javorsky G., Parsonage W. (2021). Pregnancy following heart transplantation: a single centre case series and review of the literature. Heart Lung Circ.

[50] Bedin A., Carbonnel M., Snanoudj R. (2022). Pregnancies and gynecological follow-up after solid organ transplantation: experience of a decade. J Clin Med.

[51] Kuczaj A., Pawlak S., Śliwka J., Przybyłowski P. (2022). Pregnancies after orthotopic heart transplantation: a single-center experience. Transplant Proc.

[52] Troche V., Ville Y., Fernandez H. (1998). Pregnancy after heart or heart-lung transplantation: a series of 10 pregnancies. Br J Obstet Gynaecol.

[53] Estensen M., Gude E., Ekmehag B. (2011). Pregnancy in heart- and heart/lung recipients can be problematic. Scand Cardiovasc J.

[54] Mohamed-Ahmed O., Nelson-Piercy C., Bramham K. (2014). Pregnancy outcomes in liver and cardiothoracic transplant recipients: a UK national cohort study. PLoS One.

[55] Brackley K.J. (1996). Successful pregnancy following a lung transplant. Curr Obstet Gynaecol.

[56] Paradowski L., Aris R., Novotny D., Donaldson S. (1996). Acute and chronic lung allograft rejection during pregnancy. Chest.

[57] Parry D., Hextall A., Robinson V.P., Banner N.R., Yacoub M.H. (1996). Pregnancy following a single lung transplant. Thorax.

[58] Larciprete G., Valensise H., Giannini F. (1999). Pregnancy and delivery after heart-lung transplantation: a case report and review of the literature. Ital J Gynaecol Obstet.

[59] Jongen V.H.W.M., Holm J.P., Verschuuren E.A.M., Van Der Bij W. (2000). Vaginal delivery after lung transplantation. Acta Obstet Gynecol Scand.

[60] Kruszka S.J., Gherman R.B. (2002). Successful pregnancy outcome in a lung transplant recipient with tacrolimus immunosuppression. A case report. J Reprod Med.

[61] Huang J.Q., Shahine L.K., Gupta N., Westphal L.M. (2010). Controlled ovarian hyperstimulation and gestational surrogacy in a patient with lung transplant: a case report. J Reprod Med.

[62] Divithotawela C., Chambers D., Hopkins P. (2015). Pregnancy after lung transplant: case report. Breathe (Sheffield, England).

[63] Parry D., Banner N., Hextall A., Robinson V., Yacoub M. (1997). Pregnancy following lung transplantation. Transpl Proc.

[64] Rigg C.D., Bythell V.E., Bryson M.R. (2000). Caesarean section in patients with heart-lung transplants: a report of three cases and review.

[65] Baron O., Hubaut J., Galetta D. (2002). Pregnancy and heart-lung transplantation. J Heart Lung Transpl.

[66] Gyi K.M., Hodson M.E., Yacoub M.Y. (2006). Pregnancy in cystic fibrosis lung transplant recipients: case series and review. J Cyst Fibros.

[67] Zurbano F., López F., Fornet I., de Miguel J.R., Segovia J., Ussetti P. (2012). Maternity and lung transplantation: cases in Spain. Arch Bronconeumol.

[68] Morley K., Lordan J.L., Meachery G. (2014). Pregnancy after lung and heart-lung transplantation. J Heart Lung Transpl.

[69] Greene C.L., Barr M.L., Schenkel F.A., Worrell S.G., Starnes V.A., McFadden P.M. (2014). Outcomes after pregnancy in living lobar lung transplantation. Transplantation.

[70] Bry C., Danner-Boucher I., Hubert D. (2019). Pregnancy after lung and heart-lung transplantation: a French multicentre retrospective study of 39 pregnancies. ERJ Open Res.

[71] Kulkarni H.S., Cherikh W.S., Chambers D.C. (2019). Bronchiolitis obliterans syndrome–free survival after lung transplantation: an International Society for Heart and Lung Transplantation Thoracic Transplant Registry analysis. J Heart Lung Transpl.

[72] Abdulaziz H.M., Shemies R.S., Taman M., Mosbah A., Elkannishy G. (2021). Fetal proximal and distal limb anomalies following exposure to mycophenolate mofetil during pregnancy: a case report and review of the literature. Lupus.

[73] Muduma G., Saunders R., Odeyemi I., Pollock R.F. (2016). Systematic review and meta-analysis of tacrolimus versus ciclosporin as primary immunosuppression after liver transplant. PLOS One.

[74] Webster A.C., Woodroffe R.C., Taylor R.S., Chapman J.R., Craig J.C. (2005). Tacrolimus versus ciclosporin as primary immunosuppression for kidney transplant recipients: meta-analysis and meta-regression of randomised trial data. BMJ.

[75] Coustan D.R., Lowe L.P., Metzger B.E., Dyer A.R. (2010). The Hyperglycemia and Adverse Pregnancy Outcome (HAPO) study: paving the way for new diagnostic criteria for gestational diabetes mellitus. Am J Obstet Gynecol.

[76] Metzger B.E. (2010). International Association of Diabetes and Pregnancy Study Groups Recommendations on the Diagnosis and Classification of Hyperglycemia in Pregnancy. Diabetes Care.

[77] Gestational diabetes, International Diabetes Federation (IDF).

[78] Wang W., Xie X., Yuan T. (2021). Epidemiological trends of maternal hypertensive disorders of pregnancy at the global, regional, and national levels: a population-based study. BMC Pregnancy Childbirth.

[79] Wang H., Li N., Chivese T. (2022). IDF diabetes atlas: estimation of global and regional gestational diabetes mellitus prevalence for 2021 by International Association of Diabetes in Pregnancy Study Group's Criteria. Diabetes Res Clin Pract.

[80] Pregnancy loss statistics | Tommy's (tommys.org)05/Jul/2023. Available at: https://www.tommys.org/baby-loss-support/pregnancy-loss-statistics.

[81] Stats of the States - Cesarean Delivery Rates (cdc.gov).

[82] Regitz-Zagrosek V., Roos-Hesselink J.W., Bauersachs J. (2018). 2018 ESC Guidelines for the management of cardiovascular diseases during pregnancy: The Task Force for the Management of Cardiovascular Diseases during Pregnancy of the European Society of Cardiology (ESC). Eur Heart J.

[83] Preterm Birth. Maternal and Infant Health, Reproductive Health. CDC.

[84] Majak G.B., Reisæter A.V., Zucknick M. (2017). Preeclampsia in kidney transplanted women; Outcomes and a simple prognostic risk score system. PLOS One.

[85] Cruz Lemini M.C., Ibargüengoitia Ochoa F., Villanueva González M.A. (2007). Perinatal outcome following renal transplantation. Int J Gynaecol Obstet.

