# Supplementary material for: Pregnancy outcomes and management in lung and heart transplant recipients: A systematic review
Source: JHLT Open. 2025 May 27;9:100297. doi: 10.1016/j.jhlto.2025.100297 (PMC12205842; doi:10.1016/j.jhlto.2025.100297)
Supplement: Supplementary file 1 — Supplementary material [file mmc1.docx]

**Supplementary Table S1: Search strategies for each database**

**Medline**

| 1 | exp Heart Transplantation/ |
| --- | --- |
| 2 | (cardi* adj2 transplant*).mp. |
| 3 | (heart adj2 transplant*).mp. |
| 4 | (heart adj2 graft*).mp. |
| 5 | 1 or 2 or 3 or 4 |
| 6 | exp Lung Transplantation/ |
| 7 | (lung adj2 graft*).mp. |
| 8 | (pulmonary adj2 transplant*).mp. |
| 9 | (lung adj2 transplant*).mp. |
| 10 | 6 or 7 or 8 or 9 |
| 11 | exp Pregnancy/ |
| 12 | (obstetric* adj2 outcome*).mp. |
| 13 | (birth* adj2 outcome*).mp. |
| 14 | (obstetric* adj2 complication*).mp. |
| 15 | (birth* adj2 complication*).mp. |
| 16 | (deliver* adj2 outcome*).mp. |
| 17 | (deliver* adj2 complication*).mp. |
| 18 | pregnan*.mp. |
| 19 | 11 or 12 or 13 or 14 or 15 or 16 or 17 or 18 |
| 20 | (5 or 10) and 19 |

**Embase**

| 1 | exp heart transplantation/ |
| --- | --- |
| 2 | (cardi* adj2 transplant*).mp. |
| 3 | (heart adj2 transplant*).mp. |
| 4 | (heart adj2 graft*).mp. |
| 5 | 1 or 2 or 3 or 4 |
| 6 | exp lung transplantation/ |
| 7 | (lung adj2 graft*).mp. |
| 8 | (pulmonary adj2 transplant*).mp. |
| 9 | (lung adj2 transplant*).mp. |
| 10 | 6 or 7 or 8 or 9 |
| 11 | *pregnancy/ |
| 12 | exp pregnancy outcome/ |
| 13 | (obstetric* adj2 outcome*).mp. |
| 14 | (birth* adj2 outcome*).mp. |
| 15 | (obstetric* adj2 complication*).mp. |
| 16 | (birth* adj2 complication*).mp. |
| 17 | (deliver* adj2 outcome*).mp. |
| 18 | (deliver* adj2 complication*).mp. |
| 19 | pregnanc*.mp. |
| 20 | 11 or 12 or 13 or 14 or 15 or 16 or 17 or 18 or 19 |
| 21 | (5 or 10) and 20 |

**Maternity & Infant Care Database (MIDIRS)**

| 1 | (cardi* adj2 transplant*).mp. |
| --- | --- |
| 2 | (heart adj2 transplant*).mp. |
| 3 | (heart adj2 graft*).mp. |
| 4 | 1 or 2 or 3 |
| 5 | (lung adj2 transplant*).mp. |
| 6 | (pulmonary adj2 transplant*).mp. |
| 7 | (lung adj2 graft*).mp. |
| 8 | 5 or 6 or 7 |
| 9 | pregnan*.mp. |
| 10 | (obstetric* adj2 outcome*).mp. |
| 11 | (birth* adj2 outcome*).mp. |
| 12 | (obstetric* adj2 complication*).mp. |
| 13 | (birth* adj2 complication*).mp. |
| 14 | (deliver* adj2 outcome*).mp. |
| 15 | (deliver* adj2 complication*).mp. |
| 16 | 9 or 10 or 11 or 12 or 13 or 14 or 15 |
| 17 | (4 or 8) and 16 |

**Supplementary Table S2: Inclusion and Exclusion Criteria**

| Inclusion Criteria | Exclusion Criteria |
| --- | --- |
| - Peer-reviewed publications presenting original data. - Publications presenting data related to pregnancy, and delivery outcomes of any type. - Case reports, case series, cross-sectional, case-control, cohort studies, randomized trials, or any other related study design among humans. - Conference abstracts | - Non-peer-reviewed publications (any grey literature). - Publications not presenting original data (i.e., reviews, meta-analyses, news, editorials, letters to the editor and commentaries). - Publication written not in English. - Studies on animals, cells, or other laboratory research. - Publication whose recipients receive multiorgan transplantations. |

**Supplementary table S3-1: Quality assessment tool and scoring guidance notes** (14)

**
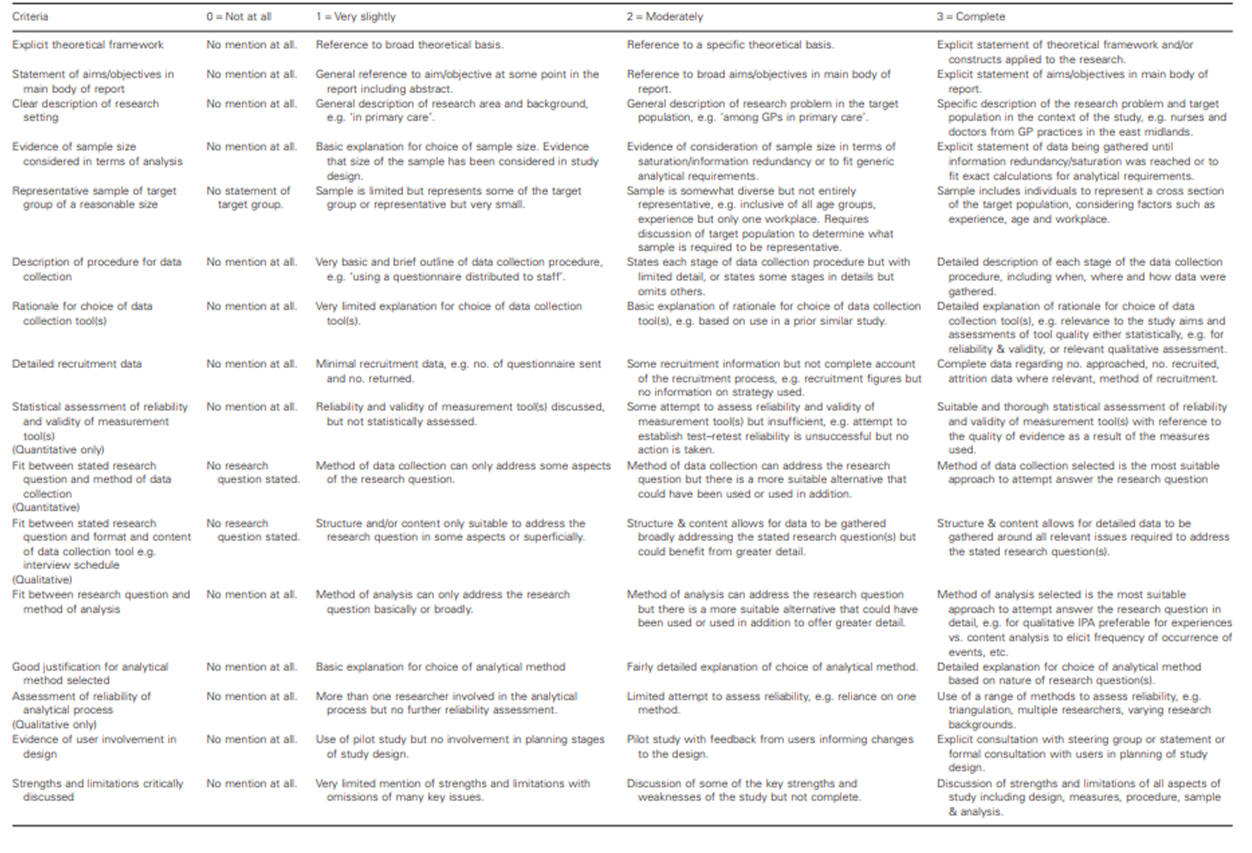
**

**Supplementary table S3-2: QATSDD scores**

|  | Bhagra 2016 | Greene 2014 | Bry 2019 | D'Souza 2018 | Dagher 2018 | Estensen 2011 | Baron 2002 | MacEra 2018 | Mohamed-Ahmed 2014 | LenkaVojtickova 2019 | Tardivo 2004 | Wasywich 2013 | Thakrar 2014 | Troche 1998 | Gyi 2006 | Parry 1997 | Haugen 1998 |
| --- | --- | --- | --- | --- | --- | --- | --- | --- | --- | --- | --- | --- | --- | --- | --- | --- | --- |
| Explicit theoretical framework | 0 | 0 | 0 | 0 | 0 | 0 | 0 | 0 | 0 | 0 | 0 | 0 | 0 | 0 | 0 | 0 | 0 |
| Statement of aims/objectives in main body of report | 3 | 2 | 2 | 3 | 3 | 3 | 2 | 2 | 2 | 3 | 3 | 3 | 3 | 2 | 1 | 1 | 2 |
| Clear description of research setting | 3 | 2 | 3 | 2 | 3 | 2 | 1 | 2 | 3 | 3 | 2 | 3 | 2 | 3 | 2 | 1 | 2 |
| Evidence of sample size considered in terms of analysis | 0 | 0 | 0 | 0 | 0 | 0 | 0 | 0 | 0 | 0 | 0 | 0 | 0 | 0 | N/A | N/A | N/A |
| Representative sample of target group of a reasonable size | 1 | 0 | 1 | 1 | 1 | 1 | 0 | 1 | 1 | 0 | 0 | 0 | 0 | 1 | N/A | N/A | N/A |
| Description of procedure for data collection | 3 | 2 | 2 | 2 | 2 | 1 | 1 | 2 | 2 | 2 | 1 | 2 | 2 | 2 | 2 | 1 | 1 |
| Rationale for choice of data collection tool | 1 | 0 | 1 | 0 | 1 | 1 | 0 | 1 | 1 | 0 | 0 | 0 | 0 | 1 | 0 | 0 | 1 |
| Detailed recruitment data | 1 | 0 | 2 | 2 | 2 | 1 | 1 | 1 | 1 | 2 | 1 | 2 | 1 | 1 | N/A | N/A | N/A |
| Statistical assessment reliability and validity of measurement tool(s) (Quantitative only) | N/A | N/A | N/A | N/A | N/A | N/A | N/A | N/A | 1 | 2 | N/A | N/A | N/A | N/A | N/A | N/A | N/A |
| Fit between stated research question and method of data collection (Quantitative) | 2 | 1 | 2 | 2 | 3 | 2 | 1 | 2 | 2 | 3 | 2 | 2 | 2 | 2 | 1 | 1 | 1 |
| Fit between stated research question and format and content of data collection tool (e.g., interview schedule) (Qualitative) | 1 | 1 | 1 | 1 | 1 | 1 | 1 | 1 | 1 | 1 | 1 | 1 | 1 | 1 | 1 | 1 | 1 |
| Fit between research question and method of analysis | 2 | 2 | 2 | 2 | 2 | 2 | 2 | 2 | 3 | 3 | 2 | 3 | 2 | 2 | 2 | 1 | 1 |
| Good justification for analytical method selected | 1 | 0 | 1 | 0 | 0 | 1 | 1 | 1 | 0 | 1 | 0 | 1 | 0 | 0 | 0 | 0 | 0 |
| Assessment of reliability of analytical process (Qualitative only) | 0 | 0 | 0 | 0 | 0 | 0 | 0 | 0 | 0 | 0 | 0 | 0 | 0 | 0 | 0 | 0 | 0 |
| Evidence of user involvement in design | 0 | 0 | 0 | 0 | 0 | 0 | 0 | 0 | 0 | 0 | 0 | 0 | 0 | 0 | 0 | 0 | 0 |
| Strengths and limitations critically discussed | 2 | 2 | 3 | 3 | 2 | 1 | 2 | 2 | 3 | 2 | 2 | 3 | 1 | 2 | 1 | 0 | 0 |
| Total | 20 | 12 | 19 | 18 | 20 | 16 | 12 | 17 | 20 | 22 | 14 | 20 | 14 | 17 | 10 | 6 | 9 |

**Supplementary table S4: Indications of transplantation (**CS/RCS and CR**)**

|  | <HTR> | | <LTR> | |
| --- | --- | --- | --- | --- |
| Indicator | Cardiomyopathy (CM) | 76 | Cystic Fibrosis | 48 |
|  | CM:31, Dilated CM:27, Peri/postpartum CM:4, Hypertrophic CM:7, Myocarditis related CM:3, Ischemic CM:1, Other CM:3 |  | Pulmonary Hypertension | 12 |
|  |  |  | Obstructive Lung Disease | 9 |
|  |  |  | Congenital Heart Disease | 8 |
|  |  |  | Interstitial Lung Disease | 7 |
|  | Congenital heart disease | 39 |  |  |
|  |  |  |  |  |
|  | <Heart/Lung Transplant Recipient> |  |  |  |
|  | Pulmonary Hypertension | 2 |  |  |
|  | Cystic fibrosis | 2 |  |  |
|  | Congenital heart disease and Pulmonary Hypertension | 6 |  |  |

Indications for HTR were mentioned in 115 of 151 heart transplant pregnancies. Indications for HLTR were mentioned in 10 of 66 heart or heart/lung or lung transplant pregnancies. ((67), (68), (69))

Indications for LTR were mentioned in 84 of 94 lung or heart/lung transplant pregnancies.

**Supplementary table S5: Countries of studies (**CS/RCS and CR**)**

|  | HTR | | LTR | |
| --- | --- | --- | --- | --- |
| Countries of studies | **8** | **US** (33) (39) (42) (43) (47) (49) (51) (53) | **6** | UK (70) (72) (75) (78) (79) (81) |
|  | **4** | **UK** (38) (45) (57) (69) | **3** | US (71) (76) (84) |
|  | **3** | Poland (41) (48) (66) | **2** | France (80) (85) |
|  |  | France (34) (65) (67) | **1** | Australia (77) |
|  | **2** | Italy (55) (62) |  | Spain (82) |
|  |  | Portugal (52) (60) |  | Canada (83) |
|  |  | New Zealand (46) (56) |  | Norway (54) |
|  |  | Australia (37) (64) |  | Italy (73) |
|  |  | Norway (54) (68) |  | Netherlands (74) |
|  | **1** | Saudi Arabia (36) |  |  |
|  |  | Brazil (44) |  |  |
|  |  | Sweden (35) |  |  |
|  |  | Japan (50) |  |  |
|  |  | Taiwan (58) |  |  |
|  |  | Czechia (63) |  |  |
|  |  | Canada (59) |  |  |
|  |  | Belgium, Canada (61) |  |  |
|  |  | Austria (40) |  |  |
|  |  | Argentina (32) |  |  |
